# The LncRNA HOTAIRM1 Promotes Tamoxifen Resistance by Mediating HOXA1 Expression in ER+ Breast Cancer Cells

**DOI:** 10.7150/jca.38728

**Published:** 2020-03-13

**Authors:** Clara Yuri Kim, Ji Hoon Oh, Ji-Yeon Lee, Myoung Hee Kim

**Affiliations:** 1 Department of Anatomy, Embryology Laboratory, Yonsei University College of Medicine, Seoul 03722, Korea.; 2 Brain Korea 21 PLUS project for Medical Science, Yonsei University College of Medicine, Seoul 03722, Korea.

**Keywords:** Breast cancer, endocrine resistance, HOTAIRM1, HOXA1, EZH2

## Abstract

Breast cancer is one of the most commonly diagnosed cancers in women worldwide. Approximately 40% of patients with breast cancer acquire endocrine resistance following therapy with tamoxifen. Many explanations for the development of endocrine resistance have been put forward, one of them being the dysregulation of long non-coding RNAs (lncRNAs). The lncRNA HOTAIRM1, known to be involved in myelopoiesis as well as transcriptional regulation of the HOXA genes in embryonic stem cells, is also expressed in breast cancer cells. This study explored the molecular mechanisms of HOTAIRM1 involved in acquired tamoxifen resistance. We showed that HOTAIRM1 and HOXA1 are concurrently up-regulated in tamoxifen-resistant MCF7 (TAMR) cells. Knockdown of HOTAIRM1 down-regulated HOXA1 expression and restored sensitivity to tamoxifen. In addition, the knockdown of HOXA1 showed similar effects, suggesting that the HOTAIRM1/HOXA1 axis regulates tamoxifen resistance. Furthermore, we showed that HOTAIRM1 directly interacts with EZH2 and prevents the PRC2 complex from binding and depositing H3K27me3 on the putative promoter of HOXA1. Together, our findings suggest that HOXA1 and its neighboring lncRNA, HOTAIRM1, might serve as potential therapeutic targets for ER+ breast cancer patients who have acquired tamoxifen resistance.

## Introduction

Breast cancer is the most commonly diagnosed cancer in women, with the estrogen-receptor positive (ER+) subtype being the most prevalent 1-3. Patients with ER+ cancer are generally treated with selective estrogen-receptor modulators (SERMs), such as tamoxifen 4,5. Patients initially respond to tamoxifen, but many eventually develop resistance to tamoxifen, ultimately leading to relapsed or metastatic breast cancer 6-8. Although there are extensive studies on the mechanisms underlying the development of tamoxifen resistance, there is still a lack of practical molecular markers to predict tamoxifen resistance and/or breast cancer recurrence. Therefore, it is imperative to identify novel biomarkers for tamoxifen resistance.

The homeobox (HOX) genes are a group of 39 genes organized into 4 different clusters: HOXA, B, C, and D. The HOX family plays key roles during embryogenesis by regulating various cellular and physiological processes 9-11. More recently, numerous *HOX* genes have been reported to act as oncogenes or tumor suppressors 12-14. For example, HOXA1 has been found to induce tumorigenesis in multiple types of cancers such as breast, lung, and gastric cancers 15-18. Nevertheless, the role of HOXA1 and its role in tamoxifen-resistant breast tumors have not yet been defined.

Emerging evidence suggests that long non-coding RNAs (lncRNAs), defined as RNA transcripts longer than 200 nucleotides that are incapable of encoding proteins, play crucial roles in transcriptional and post-transcriptional regulation, microRNA sponging, as well as chromatin remodeling 19-21. More recently, the functions and mechanisms of lncRNAs in cancer have been receiving research attention, and a number of lncRNAs have been reported to be involved in endocrine resistance, as well as breast cancer progression and metastasis 22-24. Within the HOXA cluster is a long non-coding RNA, HOTAIRM1, located between *HOXA1* and *HOXA2*. The role of HOTAIRM1 as an oncogenic factor has been described in several cancers 25,26, and the function of HOTAIRM1 as a regulator of the oncogene *HOXA1* has been previously described in glioblastoma multiforme 26. However, the regulatory role of HOTAIRM1 on *HOXA1* transcription in breast cancer, more specifically tamoxifen-resistant breast cancer, remains unknown.

In the present study, we showed that the lncRNA HOTAIRM1 is upregulated in tamoxifen-resistant breast cancer cells (TAMR), compared to levels in ER+ breast cancer cells (MCF7). HOXA1 was also shown to be upregulated in TAMR cells. Knockdown of HOTAIRM1 or HOXA1 could re-sensitize TAMR cells to tamoxifen treatment, suggesting that the HOTAIRM1/HOXA1 axis contributes to tamoxifen resistance in breast cancer. More notably, HOTAIRM1 could directly interact with EZH2 and prevent EZH2 from binding to the putative promoter of *HOXA1*, ultimately activating *HOXA1* transcription. Our results revealed a novel mechanism which demonstrates that HOTAIRM1 and HOXA1 could be promising therapeutic targets for patients with ER+ breast cancer who have acquired tamoxifen resistance.

## Materials and Methods

### Cell lines and culture

MCF7, T47D, MCF7-TAMR, and T47D-TAMR cells were used. MCF7-TAMR cells were generated as an *in vitro* model for acquired tamoxifen resistance by exposing MCF7 cells to long-term 1 μM 4-hydroxytamoxifen (Sigma, MO, USA) treatment 27. T47D and T47D-TAMR cells (T47D/S2 [152109], T47D/TR-1 [152108], and T47D/TR-2 [152110]) obtained from Ximbio were kindly provided by Dr. Mi-Ock Lee (Seoul National University). MCF7 and MCF7-TAMR cell lines were cultured in Dulbecco's modified Eagle medium (WelGENE Inc., Daegu, Korea). The medium was supplemented with heat-inactivated 10% FBS (WelGENE Inc.) and penicillin-streptomycin (WelGENE Inc.). T47D cells were maintained in RPMI 1640 (WelGENE Inc.) with the same supplementation. T47D-TAMR cells were cultured in Phenol Red-free RPMI 1640 (WelGENE Inc.) containing 2% FBS, 2 mM Glutamax (Thermo Fisher Scientific, MA, USA), 8 μg/mL human insulin (WelGENE Inc.), and 1% penicillin streptomycin. Both tamoxifen-resistant cells were cultured in medium containing 1 μM 4-hydroxytamoxifen. Cells were grown at 37 °C in a 5% CO_2_ incubator.

### Total RNA isolation and RT-PCR

Total RNA was extracted from cells using TRIzol reagent (Invitrogen, CA, USA), and cDNA was synthesized with 1 μg of total RNA using ImProm-II^TM^ Reverse Transcriptase (Promega, WI, USA). PCR amplification was performed under the following conditions: initial denaturation for 5 mins at 94 °C, followed by 27-35 cycles of 94 °C for 40 sec (depending on target gene), 58°C for 20 sec, and 72 °C for 30 sec. For quantitative PCR, a StepOnePlus Real-Time PCR System (Applied Biosystems, CA, USA) and Power SYBR Green PR Master Mix (Applied Biosystems) were used. All PCR reactions were performed in at least three independent biological replicates, and β-Actin and GAPDH were used as internal controls. PCR primers are listed in Table 1.

### siRNA and transfection

Knockdown studies were performed by transfecting TAMR cells for 48 hours with 20 nM siHOTAIRM1 or 40 nM siHOXA1 using G-fectin (Genolution, Seoul, Korea) following the manufacturer's protocol. A pool of 4 individual siRNAs targeting exons 1 and 3 of HOTAIRM1, and a single siRNA targeting exon 2 of HOXA1 were used (Supplementary Table 1). siRNAs were designed and purchased from Genolution.

### Cell proliferation assay

Relative cell proliferation was measured using a Cell Counting Kit-8 (Dojindo Molecular Technologies Inc., Kumamoto, Japan) following the manufacturer's protocol. Briefly, 7.5 × 10^3^ cells/well were plated and grown on 96-well plates, stained with 10 μL of WST-8, and reacted for 3 hours at 37 °C in a 5% CO_2_ incubator for three consecutive days. The plate was then analyzed using a Softmax Pro microplate reader (Molecular Devices, CA, USA) at an absorbance of 450 nm.

### Chromatin immunoprecipitation (ChIP) analysis

ChIP analysis was performed as previously described with minor modifications 28. Briefly, cells were fixed with 1% formaldehyde for 15 mins at room temperature, and then quenched with 2.5 M glycine. Cells were lysed on ice for 10 mins in SDS buffer containing protease inhibitors, and then sonicated with a Sonics Vibra Cell^TM^ (6 mins: pulse 10 sec, interval 10 sec) on ice. The fragmented chromatin samples were centrifuged at 8000 × *g* at 4 °C for 5 mins and supernatant was collected. The samples were pre-cleared, and then incubated overnight at 4 °C with appropriate antibodies and protein-coated A/G agarose beads (Santa Cruz, CA, USA) with gentle shaking. The immunoprecipitated eluates were reverse cross-linked and recovered through DNA purification for PCR. Anti-H3K4me3 (Abcam, ab1012), anti-H3K9ac (Abcam, ab12179), anti-H3K27me3 (Abcam, ab6002), anti-EZH2 (Cell Signaling, 5246S), and non-immune mouse IgG (Santa Cruz, sc2025) antibodies were used. ChIP-PCR primers are listed in Table 1.

### RNA immunoprecipitation (RIP) analysis

RIP analysis was performed as previously described with minor modifications 29. Briefly, 1 × 10^7^ cells/antibody were collected and lysed on ice in RIP buffer containing protease inhibitors for 10 mins, then sonicated with a Sonics Vibra Cell^TM^ (5 mins: pulse 10 sec, interval 10 sec) on ice. The lysates were centrifuged at 12,000 × *g* at 4 °C for 5 mins and the supernatant was taken. The samples were first incubated with anti-EZH2 (5246S) and non-immune mouse IgG (sc2025) antibodies at 4 °C for at least 4 hours, and then protein-coated A/G agarose beads (Santa Cruz, CA, USA) were added and incubated overnight at 4 °C with gentle shaking. On the following day, the immunoprecipitated eluates were resuspended in 1 mL TRIzol, and RNA was extracted. The desired product was detected by PCR with primers listed in Table 1.

### *In silico* analysis

The TANRIC web-accessible database was used to evaluate the correlation between HOTAIRM1 and HOXA1 mRNA expressions in patients' breast cancer tissues and various breast cancer cell lines (https://ibl.mdanderson.org/tanric/_design/basic/index.html). The TANRIC database is generated using the Cancer Genome Atlas (TCGA) data portal and the Cancer Cell Line Encyclopedia (CCLE).

### Statistical analysis

Data are expressed as the mean ± SD. Statistical differences were determined using Student's t-test for a pairwise comparison. p-values < 0.05 were considered significant.

## Results

### HOTAIRM1 is associated with tamoxifen resistance in MCF7 cells

**Figure 1:**
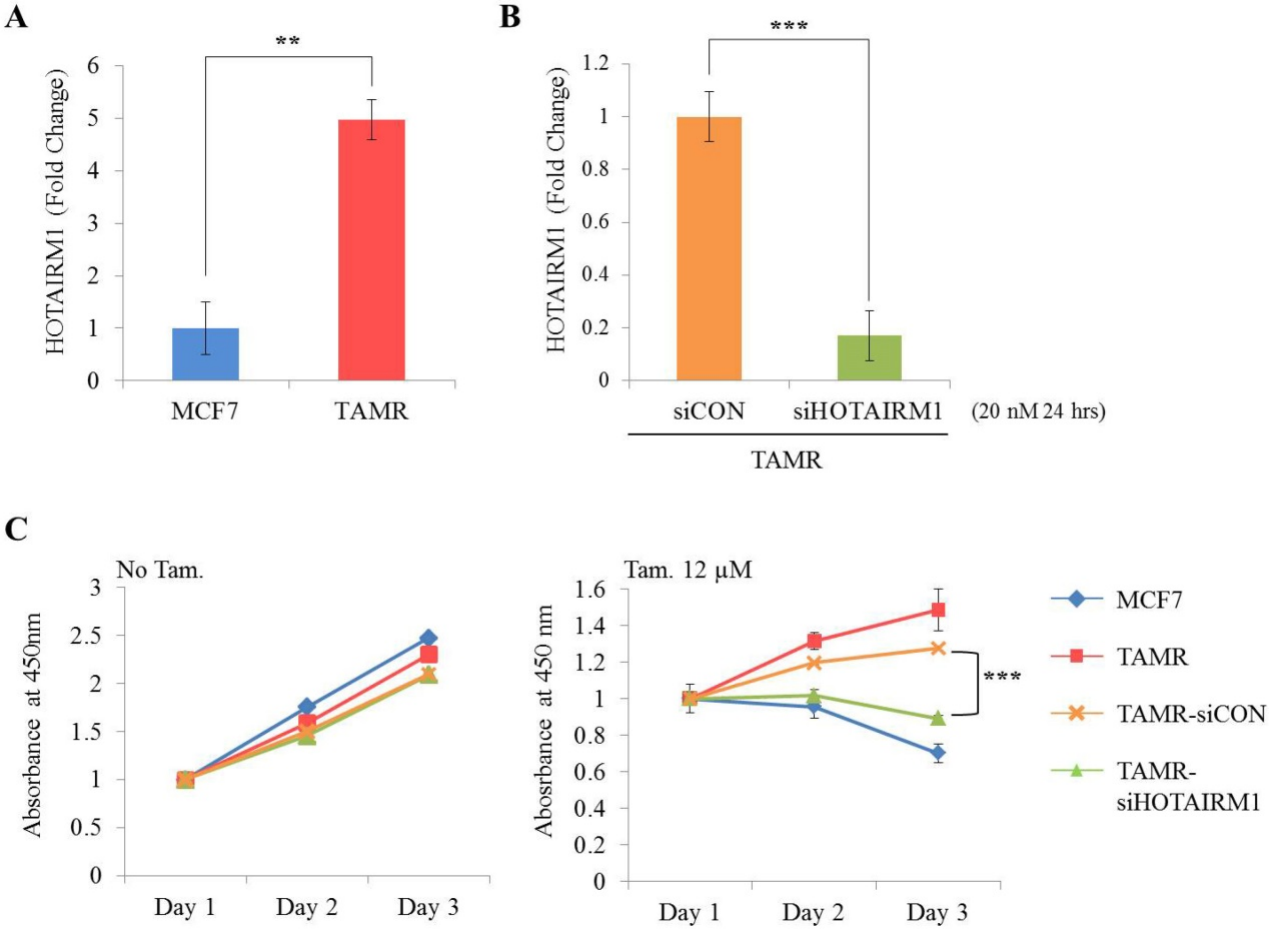
** HOTAIRM1 is associated with tamoxifen resistance in MCF7 cells. (A)** RT-qPCR analysis of HOTAIRM1 in MCF7 and tamoxifen-resistant breast cancer (TAMR) cells. **(B)** RT-qPCR analysis of HOTAIRM1 in TAMR cells transiently transfected with pooled siHOTAIRM1 for 24 hours. GAPDH was used to normalize changes in HOTAIRM1 expression levels. **(C)** Cell viability curve of cells treated with siHOTAIRM1 in the absence (No Tam.- left panel) and presence of 12 µM tamoxifen (Tam.- right panel) at days 1, 2, and 3. All experiments were performed in triplicate. *** p < 0.001 compared with siCON by Student's t-test.

First, we examined the expression level of HOTAIRM1 between ER+ breast cancer cells (MCF7 and T47D) and tamoxifen-resistant breast cancer cells (TAMR) by RT-qPCR. Our result shows that the expression of HOTAIRM1 was significantly up-regulated in TAMR cells (Fig. 1A and Fig. S1A). To explore the role of HOTAIRM1 in tamoxifen resistance, we transiently transfected TAMR cells with siHOTAIRM1, and confirmed a notable reduction in HOTAIRM1 level (Fig. 1B and Fig. S1B). The cell viability of these knockdown cells in the absence and presence of high-dose tamoxifen (12 μM) was measured and compared. In the absence of tamoxifen, knockdown of HOTAIRM1 did not result in much difference in cell proliferation in comparison to that in control cells. Interestingly, a significant re-sensitization to tamoxifen treatment was observed in HOTAIRM1-depleted TAMR cells compared to siCON-treated and parent TAMR cells (Fig. 1C and Fig. S1C). Taken together, these data suggest that HOTAIRM1 is associated with tamoxifen resistance in ER+ breast cancer cells.

### HOTAIRM1 promotes tamoxifen resistance via regulating HOXA1 expression

**Figure 2:**
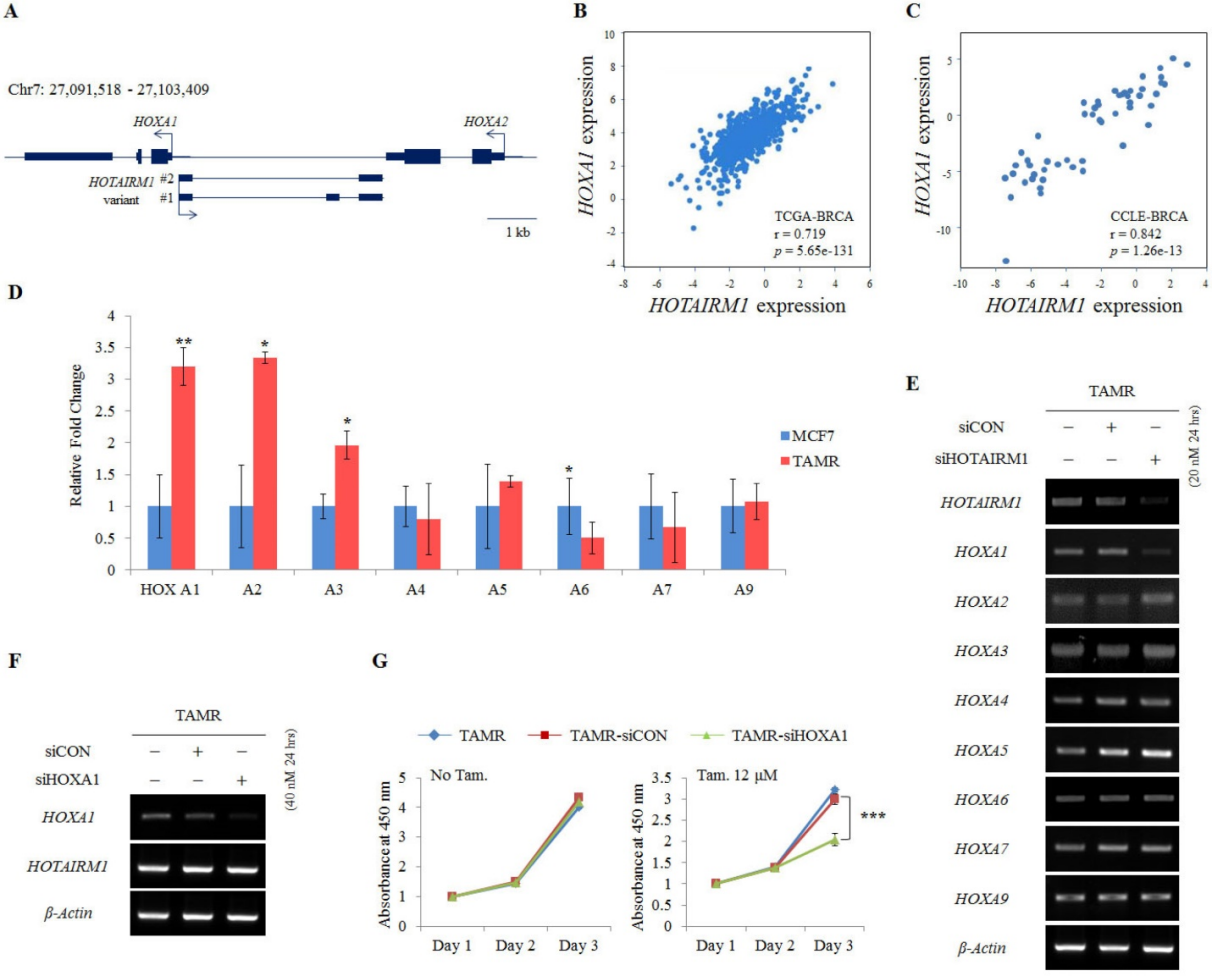
** HOTAIRM1 promotes tamoxifen resistance by regulating HOXA1 expression. (A)** Schematic depiction of the HOTAIRM1/HOXA1 locus on human chromosome 7. Boxes represent exons, lines represent introns, and arrows show the direction of transcription. **(B)** Correlation curve between HOTAIRM1 and HOXA1 expression in the TCGA breast cancer tissue database. **(C)** Correlation curve between HOTAIRM1 and HOXA1 in CCLE breast cancer cell line database. **(D)** RT-qPCR analysis of HOXA genes in MCF7 and tamoxifen-resistant breast cancer (TAMR) cells. **(E)** RT-PCR analysis of HOXA genes in TAMR cells transiently transfected with pooled siHOTAIRM1 for 24 hours. **(F)** RT-PCR of HOXA1 and HOTAIRM1 in TAMR cells transiently transfected with siHOXA1 for 24 hours. **(G)** Cell viability curve of cells treated with siHOXA1 or control siCON in the absence (No Tam.- left panel) and presence of 12 µM tamoxifen (Tam.- right panel) at days 1, 2, and 3. TAMR parent cells were included as a control without any siRNA treatment. GAPDH was used to normalize changes in the mRNA levels of interest in panel** (D)**. β-Actin was used as an internal control in panels **(E) and (F).** All experiments were performed in triplicate. *** p < 0.001 compared with siCON by Student's t-test.

HOTAIRM1 is intergenically positioned in between HOXA1 and HOXA2 at the promoter region of the HOXA1 gene (Fig. 2A). Since there are numerous reports on the function of lncRNAs as regulators of neighboring genes 22,24, we also preferentially examined the expression of genes surrounding HOTAIRM1. To confirm the association of HOTAIRM1 and HOXA1 expression in breast cancer, we first exploited TANRIC, an in silico database for lncRNAs in cancer, and found that the expression of HOTAIRM1 and HOXA1 is positively correlated in both human breast cancer tissues (TCGA, r= 0.719, p= 5.65e-131) and human breast cancer cell lines (CCLE, r = 0.842, p= 1.26e-13) (Fig. 2B and 2C).

Since the positive correlation between HOTAIRM1 and HOXA1 was confirmed through in silico analysis, we performed RT-qPCR in our model cell lines to explore this association. We confirmed that HOXA1 is most significantly overexpressed in TAMR cells. The more anterior genes such as HOXA2 and HOXA3 were also comparatively upregulated, whereas the expression levels of the other HOXA genes remained similar between MCF7 and TAMR cells (Fig. 2D). In addition, this gene expression pattern was also observed in breast cancer tissues through HOTAIRM1-mRNA correlation analysis using TCGA data retrieved from TANRIC (Fig. S2). To examine whether HOTAIRM1 functions as a regulator of the adjacent HOXA1 gene transcription in tamoxifen-resistant breast cancer cells, we treated TAMR cells with siHOTAIRM1 and examined the expression of anterior HOXA genes. Surprisingly, upon knockdown of HOTAIRM1, only the expression of HOXA1 was reduced, while the expression of HOXA2 to HOXA9 was unchanged (Fig. 2E). The exclusive down-regulation of HOXA1 upon HOTAIRM1 knockdown was also observed in T47D-TAMR cells (Fig. S3A). These data indicate that HOTAIRM1 exclusively regulates the transcription of HOXA1.

To further confirm whether tamoxifen resistance is modulated by HOTAIRM1-mediated HOXA1 transcription, we transiently transfected TAMR cells with siHOXA1 (Fig. 2F and Fig. S3B). Their proliferation rates were not altered in the absence of tamoxifen, but in the presence of tamoxifen, a significant reduction in cell viability was observed compared to that of control cells (Fig. 2G and Fig. S3C). Collectively, it seems highly likely that resistance to tamoxifen is developed through the HOTAIRM1/HOXA1 axis and that sensitivity to tamoxifen can be reverted by depleting HOTAIRM1 or HOXA1.

### HOTAIRM1 mediates HOXA1 expression through epigenetic modifications

**Figure 3:**
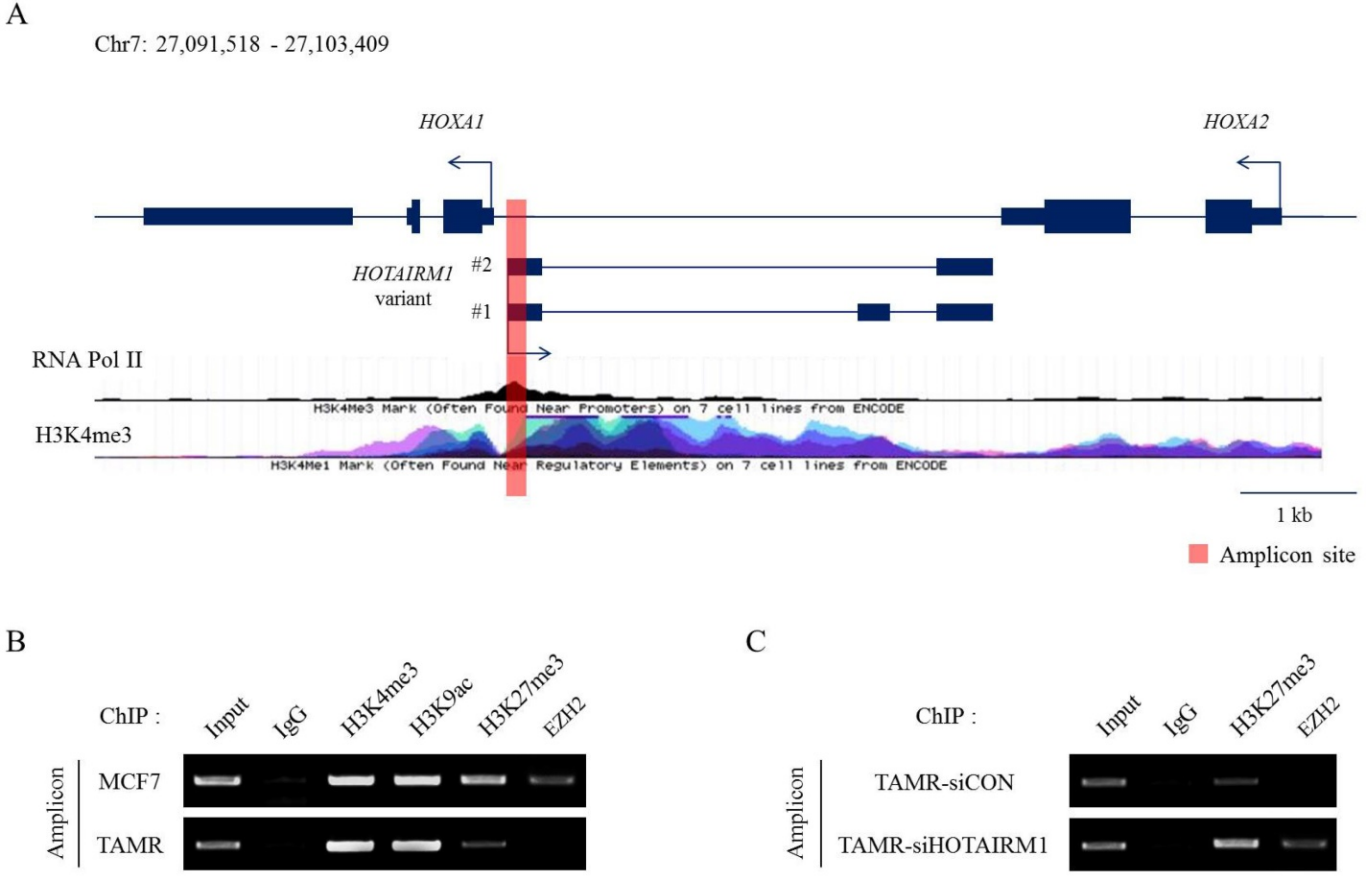
** HOTAIRM1 mediates HOXA1 expression through epigenetic modifications. (A)** Diagram representing the HOTAIRM1/HOXA1 promoter region in MCF7 cells. RNA Pol II and H3K4me3 ChIP-seq data retrieved from ENCODE. The red bar represents the amplicon site used in ChIP-PCR. **(B)** ChIP-PCR analysis of H3K4me3, H3K9ac, H3K27me3, and EZH2 in MCF7 and tamoxifen-resistant breast cancer (TAMR) cells. **(C)** ChIP-PCR analysis of H3K27me3 and EZH2 in HOTAIRM1-knockdown TAMR cells. All experiments were performed in triplicate.

Epigenetic modifications and reprogramming by chromatin remodeling factors such as the PRC2 complex are well reported in the HOX clusters. More recently, the roles of lncRNAs in recruiting epigenetic modifiers in cancer are continuously being explored 30,31. To study histone modifications and their impact on gene regulation, we first identified the putative promoter of HOXA1 by analyzing RNA Polymerase II and H3K4me3 enrichment marks (Fig. 3A). Then, we compared the distribution of histone modification patterns such as H3K4me3, H3K9ac, and H3K27me3 between MCF7 and TAMR cells at this promoter region. H3K4me3 and H3K9ac marks were enriched at similar levels in both MCF7 and TAMR cells. Nevertheless, H3K27me3 repressive marks were significantly diminished in TAMR cells compared to MCF7 cells. Moreover, EZH2, a H3K27 methyltransferase, was evidently less enriched in TAMR cells (Fig. 3B). This decrease in H3K27me3 and EZH2 explains the higher mRNA levels of HOTAIRM1 and HOXA1 in TAMR cells.

Previous studies have shown that lncRNAs are capable of sequestering epigenetic regulators from their targets by competitively binding to them. To investigate whether HOTAIRM1 has a role in regulating H3K27me3 marks by disabling the recruitment of the PRC2 complex, we performed the identical ChIP assay after knockdown of HOTAIRM1 in TAMR cells. In the presence of HOTAIRM1 (siCON sample), EZH2 binding at the putative promoter region of HOXA1 was almost undetectable; however, in HOTAIRM1-depleted TAMR cells, EZH2 marks returned, implicating that HOTAIRM1 interferes with the recruitment of the PRC2 complex to the promoter region. Along with EZH2, H3K27me3 marks were increased in the HOTAIRM1-depleted TAMR cells, further clarifying the role of HOTAIRM1 as a competitive interacting partner of EZH2 (Fig. 3C).

### HOTAIRM1 directly interacts with EZH2 and hinders the deposition of H3K27me3 marks at the putative HOXA1 promoter

**Figure 4:**
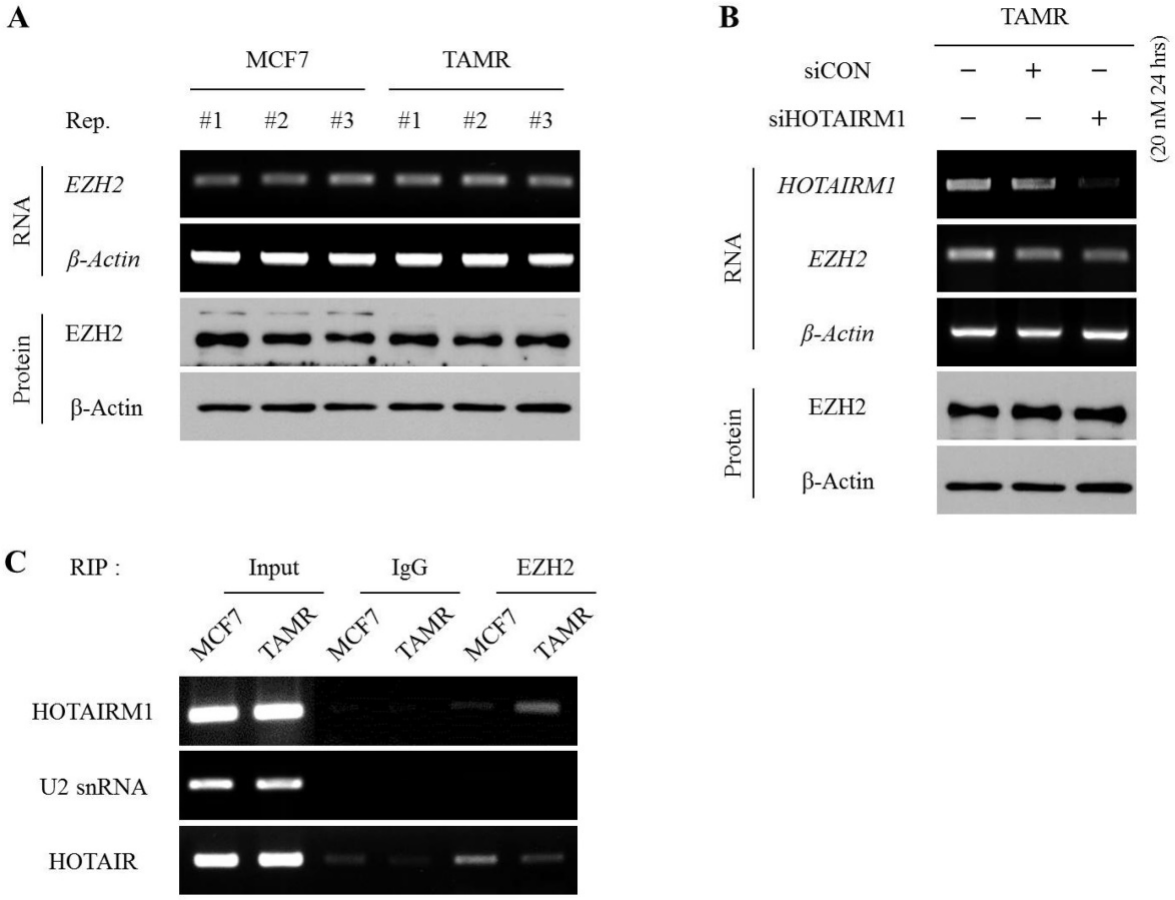
** HOTAIRM1 directly interacts with EZH2 and hinders the deposition of H3K27me3 marks at the putative HOXA1 promoter. (A)** RT-PCR and western blot showing RNA and protein levels of EZH2 in MCF7 and tamoxifen-resistant breast cancer (TAMR) cells. **(B)** RT-PCR and western blot showing RNA and protein levels of EZH2 in HOTAIRM1-knockdown TAMR cells. β-Actin was used as an internal control. **(C)** RIP-PCR analysis for the interaction between HOTAIRM1 and EZH2 in MCF7 and TAMR cells. U2snRNA was used as a negative control, and HOTAIR was used as a positive control. All experiments were performed in triplicate.

To define whether HOTAIRM1 regulates EZH2 expression at the transcriptional level or affects the deposition of methyl marks at H3K27 by interacting with pre-existing EZH2, we first examined and compared the mRNA and protein expression levels of EZH2 between MCF7 and TAMR cells. The mRNA and protein levels of EZH2 were comparable between the two cell lines (Fig. 4A). Likewise, even upon siHOTAIRM1 treatment, the expression levels of EZH2 in both mRNA and protein forms were unchanged, implying that HOTAIRM1 does not have any effect on regulating the expression of EZH2 (Fig. 4B). Therefore, we hypothesized that HOTAIRM1 may have a role in modulating the binding affinity of EZH2 at the putative HOXA1 promoter region. To validate our hypothesis, we performed a RIP assay to see whether HOTAIRM1 and EZH2 form a complex, and if there is any variance in the interaction frequency between MCF7 and TAMR cells. Remarkably, HOTAIRM1 and EZH2 formed direct interactions in TAMR cells, while there was no presence of direct binding between HOTAIRM1 and EZH2 in MCF7 cells (Fig. 4C). These results indicate that in tamoxifen-resistant breast cancer cells, HOTAIRM1 complexes with EZH2, preventing the enzyme from binding to the putative promoter of HOXA1 to deposit repressive H3K27me3 marks.

## Discussion

Here, we propose that HOTAIRM1, a highly expressed lncRNA in tamoxifen-resistant breast cancer cells, is a novel marker for tamoxifen resistance. The overexpressed HOTAIRM1 results in the up-regulation of its target gene, HOXA1, and is accompanied by an increase of tamoxifen resistance. Conversely, upon transient knockdown of HOTAIRM1, the expression of HOXA1 was concomitantly reduced, and sensitivity to tamoxifen treatment was restored. More importantly, we discovered that HOTAIRM1 inhibits the PRC2 complex from binding to the HOXA1 promoter region in TAMR cells by forming a direct interaction with EZH2, resulting in the higher expression of HOXA1.

Previous studies have identified HOTAIRM1 as a key lncRNA biomarker in several solid tumors, as well as in leukemia, by acting as tumor suppressors or oncogenes. For example, in hepatocellular carcinoma and gastric cancer, HOTAIRM1 prevents cancer progression by suppressing signaling cascades such as the Wnt and the PI3K/AKT pathways 32,33. On the other hand, high expression of HOTAIRM1 has been associated with oncogenicity in pancreatic ductal carcinoma by promoting proliferative and migratory abilities 25. In addition, the role of HOTAIRM1 in occluding epigenetic factors like G9a and EZH2, and reducing repressive histone enrichments have been reported in glioblastoma multiforme 26. Nevertheless, no study has been conducted to investigate the role of HOTAIRM1 in drug resistance in breast cancer.

Our study focused on the mechanism by which HOTAIRM1 regulates HOXA1 in tamoxifen-resistant breast cancer cells. We showed that HOTAIRM1 hinders the catalytic activity of EZH2, an enzymatic component of the PRC2 complex, at the putative promoter region of HOXA1, resulting in demethylation of H3K27 in TAMR cells. More specifically, our RIP results further confirmed that HOTAIRM1 impedes the function of EZH2 through direct interaction, preventing EZH2 from binding and depositing methyl marks on H3K27 at the HOXA1 promoter region. To further validate the clinical relevance of HOTAIRM1 and HOXA1, we analyzed a publicly available clinical in silico dataset comparing 60 paired primary ER+ breast cancer patients to recurred cancer patients following tamoxifen mono-therapy for 5 years (GSE1379). Similar to our *in vitro* results, the expression of HOTAIRM1 and HOXA1 were up-regulated in the recurred cancer patient group (Fig. S4), supporting our conclusion that HOTAIRM1 and HOXA1 are prospective indicators for tamoxifen resistance.

HOTAIRM1 has 2 transcript variants with 2 or 3 exons, with the first and last exons being shared. This lncRNA seems to undergo alternative splicing, and a previous study reporting on the role of HOTAIRM1 in the regulation of temporal collinear activation of HOXA genes in embryonic stem cells showed the major isoform to be the spliced form with all 3 exons 34. On the contrary, in our breast cancer model system, we found that the spliced form with exons 1 and 3 was the predominant form. Similarly, in acute myeloid leukemia (AML), the spliced form containing exons 1 and 3 was shown to be more prominent 34,35. In both our system and the AML model, the spliced form containing exon 2 could not be detected. This phenomenon would be of great interest to investigate among different cell and tissue types, as different isoforms may have different cellular and physiological functions.

Our study reveals for the first time that HOTAIRM1 is not only involved in cancer progression, but is also a marker of tamoxifen resistance in breast cancer. HOTAIRM1 regulates HOXA1, and by doing so, contributes to an acquired tamoxifen resistance. The overexpression of HOTAIRM1 and/or HOXA1 could be potential biomarkers for tamoxifen resistance in patients receiving or that have received adjuvant tamoxifen therapy. Moreover, inhibitors of HOTAIRM1 and/or HOXA1 represent a possible therapeutic strategy to restore sensitivity in patients that have acquired tamoxifen resistance. Further exploration of the molecular mechanisms of the HOTAIRM1/HOXA1 axis may provide more insight in the pathogenesis of breast cancer.

## Supplementary Material

Supplementary figures and tables.

## Figures and Tables

**Table 1:** List of primers used for PCR and ChIP-PCR

Primers	Forward primer (5'-3')	Reverse primer (5'-3')
h-HOTAIRM1	GGAGCGAAGAAGAGCAAAAGC	CAGACCTCTCGCCAGTTCAT
h-HOXA1	TCATATGGACAGGAGCACCA	TGACCCAGGTAGCCGTACTC
h-HOXA2	CCTTTTGAGCAGACCATTCC	AGGGATTCTTTGTGGCTGAG
h-HOXA3	CTGCTCAACTCACCCACAGT	GCTTTCGCCTGAGCTGGA
h-HOXA4	CCCACCTTCCTTACCTCCTC	CCCAGAAGGGGACAACAGTA
h-HOXA5	ACCCACATCAGCAGCAGAGA	GGCCGCCTATGTTGTCAT
h-HOXA6	TTTTCTCCCGAGCAGCAGTA	ATGGCTCCCATACACAGCAC
h-HOXA7	ATGTACGCCCTGATGTTTCC	ACAGGAGATGAAGGGCATTG
h-HOXA9	CCACGCTTGACACTCACACT	AGTTGGCTGCTGGGTTATTG
HOXA1 Promoter ChIP	CAAAAGTTTGCCGGCTTCCG	TCCAAATCGGCCTTTGCAGT
β-Actin	CATGTTTGAGACCTTCAACACCCC	GCCATCTCCTGCTCGAAGTCTAG
GAPDH	TATAAATTGAGCCCGCAGCC	CCCAATACGACCAAATCCGTTG
