# Supplementary material for: The LncRNA HOTAIRM1 Promotes Tamoxifen Resistance by Mediating HOXA1 Expression in ER+ Breast Cancer Cells
Source: J Cancer. 2020 Mar 13;11(12):3416–23. doi: 10.7150/jca.38728 (PMC7150441; doi:10.7150/jca.38728)
Supplement: Supplementary file 1 — Supplementary figures and tables. [file jcav11p3416s1.pdf]

Supplementary Information

The LncRNA HOTAIRM1 Promotes Tamoxifen Resistance by Mediating HOXA1 Expression in ER+ Breast Cancer Cells

Clara Yuri Kim[1,2], Ji Hoon Oh[1], Ji-Yeon Lee[1,*], and Myoung Hee Kim[1,2,*]

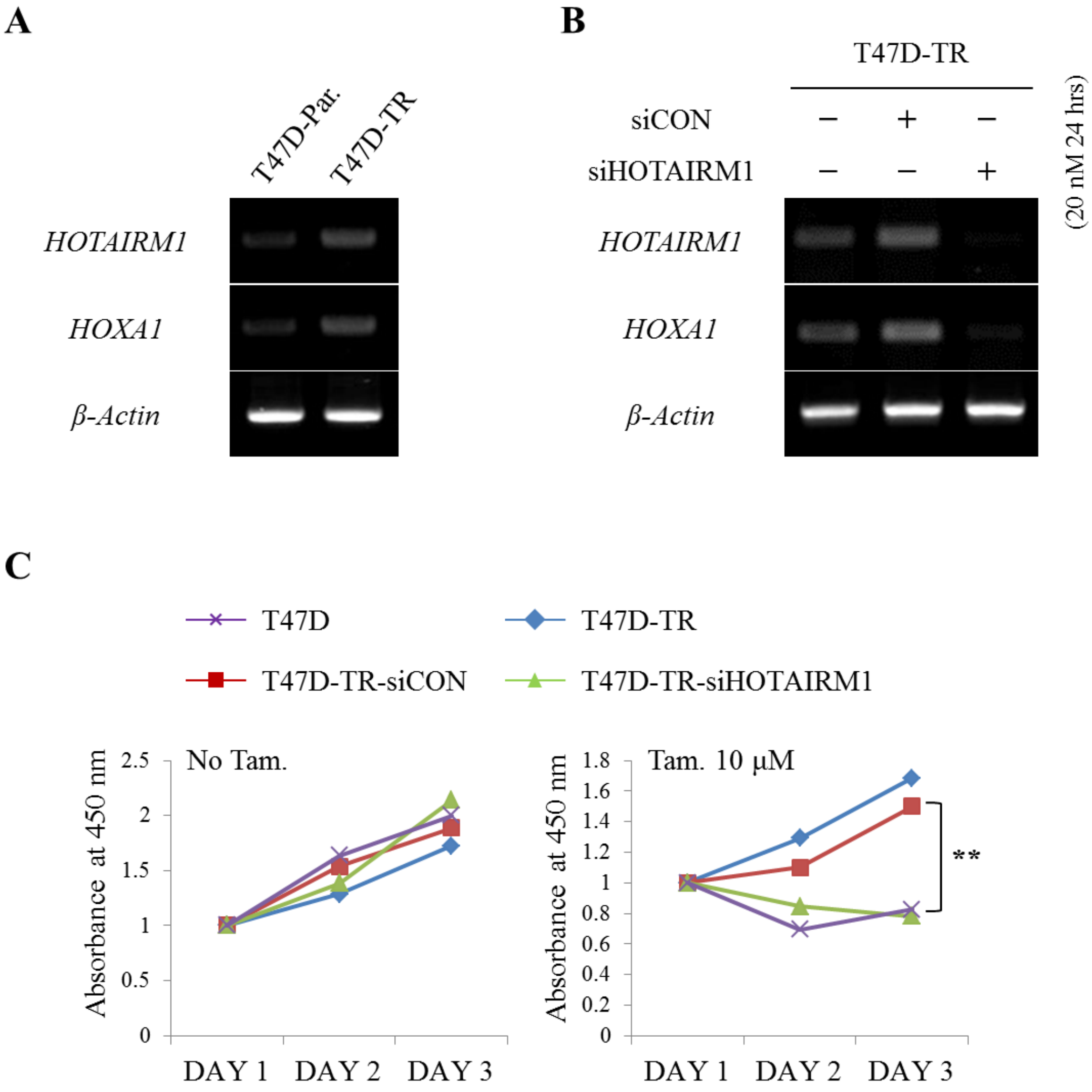

Supplementary Figure S1. HOTAIRM1 is associated with tamoxifen resistance in T47D cells. (A) RT-PCR analysis of HOTAIRM1 in T47D and tamoxifen-resistant T47D (T47D-TR) cells. (B) RT-PCR analysis of HOTAIRM1 and neighboring *HOXA1* in T47D-TR cells transiently transfected with pooled siHOTAIRM1 for 24 hours. β-Actin was used as an internal control. (C) Cell viability of cells treated with siHOTAIRM1 in the absence (No Tam.– left panel) and presence of 10 μM tamoxifen (Tam.– right panel) at days 1, 2, and 3. All experiments were performed in triplicate. *** $p < 0.001$ compared with siCON by Student's *t*-test.

HOTAIRM1: corr. mRNA

Show 10 entries Search:

Cancer	Position/Annotation	Gene Symbol	Correlation	P-value	Plot
TCGA-BRCA	ENSG00000233429.5	HOXA1	0.719	5.65e-131	
TCGA-BRCA	ENSG00000233429.5	HOXA3	0.684	0	
TCGA-BRCA	ENSG00000233429.5	HOXA4	0.645	0	
TCGA-BRCA	ENSG00000233429.5	HOXA2	0.637	3.88e-94	
TCGA-BRCA	ENSG00000233429.5	HOXA5	0.618	2.25e-87	
TCGA-BRCA	ENSG00000233429.5	BOC	0.585	0	
TCGA-BRCA	ENSG00000233429.5	HOXA7	0.578	0	
TCGA-BRCA	ENSG00000233429.5	PLAGL1	0.576	0	
TCGA-BRCA	ENSG00000233429.5	DMD	0.57	0	
TCGA-BRCA	ENSG00000233429.5	KLHL29	0.57	0	
Cancer	Position/Annotation	Gene Symbol	Correlation	P-value	Plot

Showing 1 to 10 of 524 entries First Previous 1 2 3 4 5 Next Last

Supplementary Figure S2. Positive correlation between HOTAIRM1 and anterior HOXA cluster genes in breast cancer tissues retrieved from TANRIC.

A

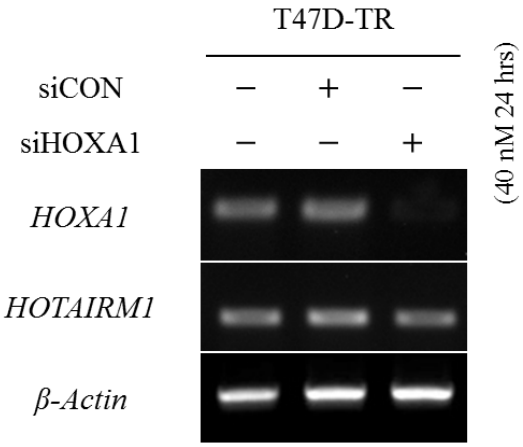

B

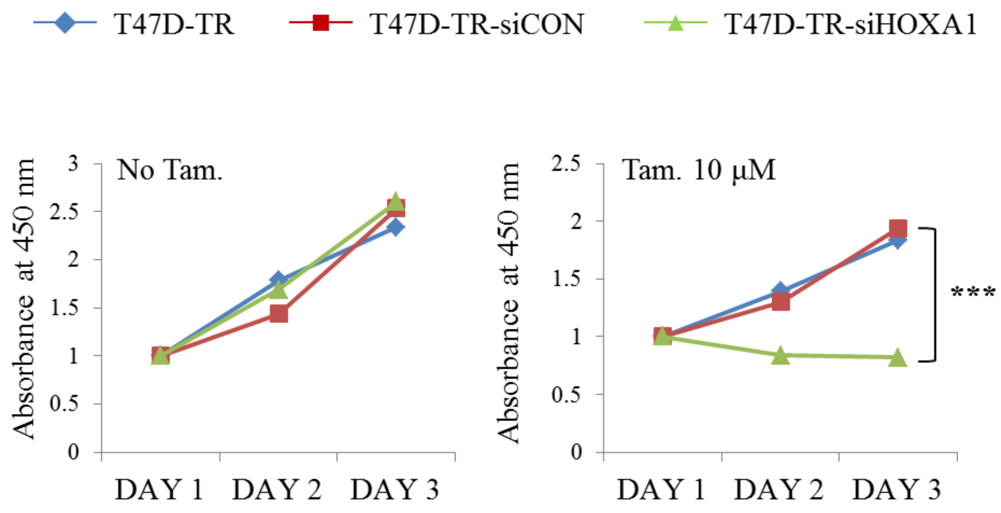

Supplementary Figure S3. HOTAIRM1 promotes tamoxifen resistance by regulating HOXA1 expression in T47D cells. (A) RT-PCR of HOTAIRM1 and *HOXA1* in T47D-TR cells transiently transfected with siHOXA1 for 24 hours. β-Actin was used as an internal control. (B) Cell viability curve of cells treated with siHOXA1 or control siCON in the absence (No Tam.– left panel) and presence of 10 μM tamoxifen (Tam.– right panel) at days 1, 2, and 3. All experiments were performed in triplicate. *** $p < 0.001$ compared with siCON by Student's *t*-test.

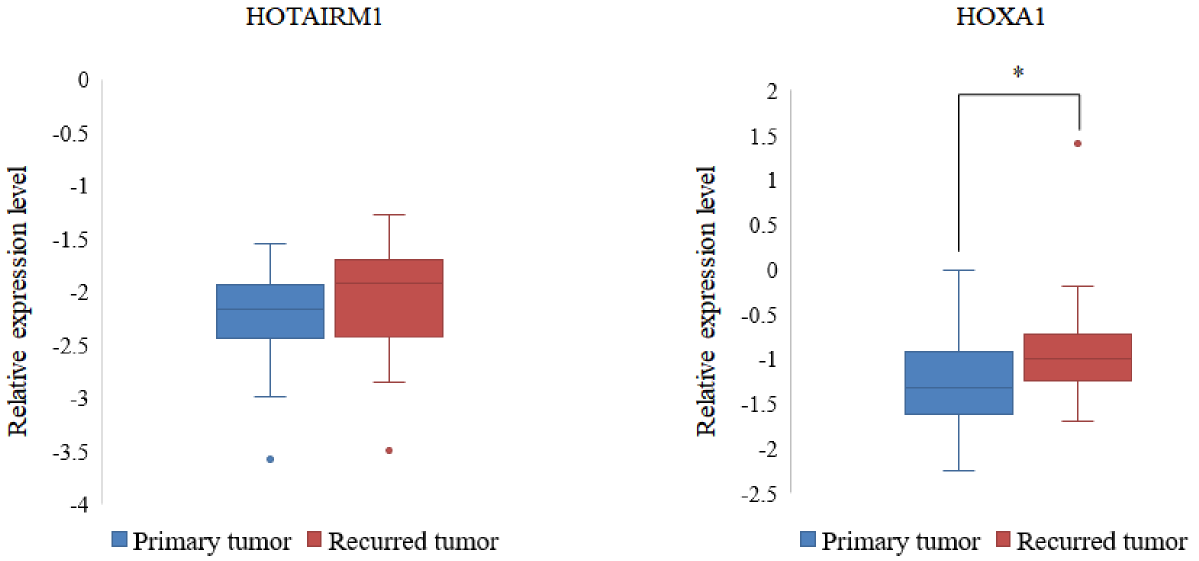

Supplementary Figure S4. Box plot of HOTAIRM1 and HOXA1 expressions in primary ER+ breast tumors and recurred tumors following tamoxifen mono-therapy for 5 years. Data were retrieved from GSE1379, a publicly available gene expression profiling data from 60 paired patients. * $p < 0.05$ compared with primary tumor by Student's *t*-test.

Supplementary Table 1. List of siRNA sequences.

Primers	Sense (5'-3')	Antisense (5'-3')
HOTAIRM1-1	CCGUUCAAUGAAAGAUGAAUU	UUCAUCUUUCAUUGAACGGUU
HOTAIRM1-2	AGAAACUCCGUGUUACUCAUUUU	AAUGAGUAACACGGAGUUUCUUU
HOTAIRM1-3	GUUUCUGUAGGCACUUUAUUU	AUAAAGUGCCUACAGAAACUU
HOTAIRM1-4	CCUCUAUUACCAAUUUAAAUU	UUUAAAUUGGUAAUAGAGGUU
HOXA1	GAGGAAUCCUCAGAGAAGUUU	ACUUCUCUGAGGAUUCCUCUU
Control	CCUCGUGCCGUUCCAUCAGGUAGUU	CUACCUGAUGGAACGGCACGAGGUU
